# Daily fluctuation of genus *Prevotella* in porcine colon under *ad libitum* feeding and its association with nutrient substrates

**DOI:** 10.3389/fmicb.2025.1688301

**Published:** 2025-09-26

**Authors:** Yue Li, Jinwei You, Yiyan Liao, Dongfang Wang, Hongyu Wang, Yong Su

**Affiliations:** ^1^Laboratory of Gastrointestinal Microbiology, Jiangsu Key Laboratory of Gastrointestinal Nutrition and Animal Health, College of Animal Science and Technology, Nanjing Agricultural University, Nanjing, China; ^2^Department of Laboratory Animal, Jinling Hospital, Affiliated Hospital of Medical School, Nanjing University, Nanjing, China; ^3^College of Animal Science, Anhui Science and Technology University, Chuzhou, China

**Keywords:** daily fluctuation, nutrient substrate, porcine, *Prevotella*, gut microbiota

## Abstract

The circadian rhythms of the gut microbiota are biologically significant for the host. However, the association between fluctuations in the relative abundance of the microbiota and nutrient substrates in the gut remains incompletely understood. Using swine as a model, this study employed continuous sampling at 9 time points over 24 h via a colonic T-shaped fistula. It investigated the temporal dynamics of nutrient substrates and *Prevotella* abundance in the colon of pigs over a 24-h period and further explored dynamic interactions among KEGG level-3 pathways, genes, and *Prevotella* using metagenomic approaches. Results revealed a significant 24-h periodicity in *Prevotella* abundance, peaking at T06–T09 and declining to minimal levels at T18–T21, with the nadir at T18. Dynamic correlation network analysis uncovered significant temporal associations between *Prevotella* rhythms and nutrient substrates: negative correlations with true protein (TP) and ammonia nitrogen (NH₃-N), in contrast to positive correlations with starch and cellulose, exhibiting time lags ranging from −2 to 4 h. *Prevotella copri* exhibited high relative abundance and pronounced daily fluctuations, while *Prevotella* sp. *MGM2* showed relatively high abundance but lacked daily fluctuations. Furthermore, differences existed in the dynamic correlations of genes and KEGG level-3 metabolic pathways of these two *Prevotella* species with nutrient substrates. The results revealed that the two *Prevotella* species in the colon exhibited different response strategies to nutrient substrates: *Prevotella copri* likely adopted a “rhythmic substrate-responsive strategy,” while *Prevotella* sp. *MGM2* followed a “sustained response strategy,” which may explain their distinct daily fluctuations.

## 1 Introduction

The mammalian gastrointestinal tract hosts a vast consortium of trillions of microorganisms that engage in symbiotic interactions with the host, critically sustaining intestinal homeostasis (Hooper and Macpherson, 2010). Functioning as a dynamic and intricate ecosystem, the gut microbiota serves crucial roles in modulating host metabolism, orchestrating immune responses, and facilitating nutrient assimilation (Frazier and Chang, 2020). Emerging evidence highlights that microbial communities exhibit intrinsic daily oscillations, which are temporally synchronized with the host’s endogenous circadian clock and feeding fasting cycles. These rhythmic fluctuations are not merely passive adaptations but actively engage in bidirectional crosstalk with host physiology, exerting systemic influences on energy homeostasis, inflammatory signaling, and behavioral outputs (Lotti et al., 2023). Given the high similarity between pigs and humans in genetic information, anatomical structure, dietary patterns, physiological functions, and other aspects, this study uses pigs as a model, aiming for the research findings on porcine gut microbiota to provide an important reference for exploring the mechanisms of human gut microbiota (Xiao et al., 2016).

In rodent models, gut microbial oscillations are modulated by daily behavioral patterns, feeding schedules, and sex-specific factors, exhibiting phylum-level differences, with Bacteroidota (particularly *Prevotella*) demonstrating more robust rhythmicity in female mice compared to males (Thaiss et al., 2014). Perturbations in microbial rhythms, often induced by erratic feeding regimens or genetic disruptions of circadian regulators, are strongly associated with metabolic dysregulation and compromised gut integrity. Therefore, deciphering the regulatory mechanisms governing microbial daily dynamics is critical for optimizing host-microbe symbiosis and improving health outcomes (Gutierrez Lopez et al., 2021). As the most abundant genus within the Bacteroidota phylum, *Prevotella* is widely distributed across multiple human body sites (e.g., oral cavity, gut, skin, vagina), animal hosts (e.g., ruminants, swine), and natural environments. Its genome size varies significantly (2.37–4.26 Mb), with high functional gene diversity, particularly in carbohydrate metabolism (Tett et al., 2021). Studies have demonstrated that the abundance of *Prevotella* in individuals consuming non-Westernized diets (rich in plant-based fibers such as grains, root vegetables, and unprocessed plants) exhibit significantly higher *Prevotella* abundance than westernized populations, where *Bacteroides* dominates (Accetto and Avguštin, 2021). Under high-fiber dietary conditions, the gut microbiota exhibits a marked enrichment of Bacteroidota (primarily *Prevotella*) and a reduction in Firmicutes (De Filippo et al., 2010). Our prior research in swine models demonstrated that nutrient substrates critically influence diurnal oscillations and functional dynamics of colonic microbiota. Notably, these microbial fluctuations correlate closely with oscillations in nutrient substrates and their metabolites, with the most pronounced rhythmic shifts observed in Bacteroidota and Firmicutes (Wang et al., 2023).

Among gut microbiota genera with pivotal functional roles, *Prevotella*, particularly *Prevotella copri*, dominates the swine gut, especially in high dietary fiber environments (Abdelsalam et al., 2023). *Prevotella copri* specializes in degrading non-starch polysaccharides (NSPs), including hemicelluloses (e.g., xylan, arabinoxylan), cellulose, and pectin. By breaking down complex plant polysaccharides, *Prevotella copri* generates short-chain fatty acids (SCFAs), which provide energy to the host and enhance intestinal barrier function (de Oliveira Melo et al., 2024). Intriguingly, *Prevotella copri* abundance in the swine gut exhibits significant daily fluctuations. Studies reveal its association with reduced fasting blood glucose, improved glucose tolerance, and lowered insulin resistance indices, suggesting its potential role as a rhythm-sensitive probiotic (Chang et al., 2019; Yang et al., 2024). Notably, the robust oscillations of *Prevotella copri* align with fluctuations in intestinal nutrient substrates, implying time-dependent functional roles in nutrient processing. However, the drivers of its rhythmic behavior and the functional significance of this temporal regulation remain unclear, warranting in-depth mechanistic exploration.

A key unresolved question is how nutrient substrates temporally shape the circadian programs of microorganisms. In previous studies, we used pigs as a model, analyzed the dynamic correlations between colonic nutrient substrates and gut microbiota over 48 h, and identified daily fluctuations therein (Wang et al., 2023). In this paper, we use *Prevotella* with strong rhythmicity as the entry point to analyze its nutrient substrate response patterns within 24 h. While feeding-fasting cycles and dietary composition are recognized as key modulators of microbial activity, the precise mechanisms underlying the rhythmic interaction between dietary components and *Prevotella* remain unclear. This study aimed to analyze the dynamic relationship between dietary components and *Prevotella* abundance in the colon across circadian timescales and further explore the genetic-level differences among *Prevotella* species with distinct daily fluctuations.

## 2 Materials and methods

### 2.1 Animals, experimental design and sampling

Eight healthy castrated male pigs (Duroc × Landrace × Yorkshire; mean body weight ± SE: 57.03 ± 1.78 kg) were anesthetized preoperatively with 3% phenobarbital sodium solution (30 mg/kg) via ear vein injection. Each pig was surgically implanted with a T-shaped cannula (inner diameter: 15 mm, length: 82 mm, wing: 10 mm) in the proximal colon, followed by intramuscular injection of antibiotics (Sodium Ceftiofur) for 7 days to prevent infection (Rivera-Zavala et al., 2011). Studies have shown that after ceftiofur administration, there are no significant changes in intestinal microbiota at the phylum level, but the community structure (Yue & Clayton index) has recovered to near baseline levels after 14 days (Costa et al., 2015). Pigs were fed under a 12-h light–dark cycle (lights on: 07:00 a.m. to 07:00 p.m.) with free access to commercial diet (Table 1) and water for 15 days. On day 16, five healthy pigs were randomly selected for colonic digesta collection starting at 06:00 a.m., with samples collected every 3 h over a 24-h period: T06 (06:00 a.m.), T09 (09:00 a.m.), T12 (12:00 p.m.), T15 (03:00 p.m.), T18 (06:00 p.m.), T21 (09:00 p.m.), T24 (12:00 a.m.), T27 (03:00 a.m.) and T30 (06:00 a.m.). The digesta samples were immediately flash-frozen in liquid nitrogen and stored at −80 °C for further metagenomic sequencing and nutrient substrate analysis. Under ad libitum feeding, the collected samples at nine time points (*n* = 5) served as controls for each other.

**Table 1:** Ingredient composition and calculated nutritional level of the experimental diet (as-fed basis).

Ingredient	Percentage (%)	Calculated nutrients	Compositions (%)
Corn	70.0	Digestive energy (MJ/kg)	14.6
Soybean meal	18.0	Crude protein	16.0
Wheat bran	6.50	Lysine	1.23
Soybean oil	1.90	Methionine+Cystine	0.70
Lysine	0.69	Threonine	0.79
Methionine	0.24	Tryptophan	0.22
Threonine	0.30		
Tryptophan	0.07		
Calcium hydrogen phosphate	0.45		
Stone powder	0.50		
Salt	0.30		
Multivitamins^1^	0.03		
Minerals^2^	0.20		
Choline chloride (50%)	0.12		
Zeolite powder	0.60		
Antioxidant	0.10		
Total	100.0		

1 For multivitamins, the diet provides per kilogram: vitamin A (VA) 11,000 IU, vitamin D3 (VD3) 1,000 IU, vitamin E (VE) 16 IU, vitamin K1 (VK1) 1 mg, vitamin B1 (VB1) 0.6 mg, vitamin B2 (VB2) 0.6 mg, d-pantothenic acid 6 mg, nicotinic acid 10 mg, vitamin B12 (VB12) 0.03 mg, folic acid 0.8 mg, and vitamin B6 (VB6) 1.5 mg.

2 Each kilogram of diet contains the following mineral supplements: iron (Fe) 165 mg, zinc (Zn) 165 mg, copper (Cu) 16.5 mg, manganese (Mn) 30 mg, cobalt (Co) 0.15 mg, iodine (I) 0.25 mg, and selenium (Se) 0.25 mg.

### 2.2 DNA extraction and metagenomic sequencing

Total genomic DNA of colonic chyme bacteria was extracted using the cetrimonium bromide (CTAB) method (Dai et al., 2010). A total of 0.5 μg DNA was used for metagenomic sequencing. Raw sequence reads were quality-filtered using Trimmomatic to remove low-quality reads and adapter contaminants (Bolger et al., 2014). Host genome contamination and low-quality data were further eliminated using the BWA-MEM algorithm (parameters: -M -k 32 -t 16)^1^ after quality control. Clean reads were assembled into contigs using MetaGenome Assembler MEGAHIT (v1.1.3) with the parameter ‘--min-contig-len 500’ (Li et al., 2015). Open reading frames (ORFs) were predicted from the assembled contigs using Prodigal (v2.6.3) (Wu and Singer, 2021). CD-HIT (v4.6) was employed for clustering ORFs into a non-redundant gene catalog with parameters ‘-n 9 -c 0.95 -G 0 -M 0 -d 0 -aS 0.9 -r 1’, where the longest sequence in each cluster was designated as the representative sequence (Fu et al., 2012). Gene abundance across samples was quantified using Salmon (v0.12.0), obtaining read counts per gene. Relative abundance was normalized as transcripts per kilobase per million mapped reads (TPM) (Patro et al., 2017). This non-redundant gene set was then searched against the Kyoto Encyclopedia of Genes and Genomes (KEGG) databases using BLASTX to identify proteins and annotate their functions. Based on the KO (KEGG Orthology) results, the specific functions and metabolic pathways were obtained by mapping the annotated genes to the KEGG Pathway Database. The gene abundance was calculated finally using the following Equations 1–5:


(1) Ab(S)=Ab(U)+Ab(M)

(2) $$\mathrm{Ab}(U)=\sum_{i=1}^{M}1/1$$

(3) $$\mathrm{Ab}(M)=\sum_{i=1}^{M}(\mathrm{CO}\times 1)/1$$

(4) $$\mathrm{CO}=\frac{\mathrm{Ab}(U)}{\sum_{i=1}^{N}\mathrm{Ab}(U_{i})}$$

(5) $$\mathrm{TPM}=\frac{(\text{counts}(t)/\text{gene}(\text{length}(t)))\times 10^{6}}{\sum_{1}^{n}\text{counts}/\text{gene length}}$$

### 2.3 Measurement of nutrient substrates in the colon

Quantitative analysis of colonic carbon (starch, cellulose) and nitrogen (TP, NH3-N) substrates was performed using standardized biochemical assays. Starch content was determined via enzymatic hydrolysis with the Solarbio BC0700 Detection Kit (Beijing Solarbio Science & Technology Co., Ltd.), while cellulose quantification employed the Solarbio BC4280 Kit, both following the manufacturer’s protocols (Zhuang et al., 2019). TP concentration was assessed through the Coomassie Brilliant Blue G-250 binding method using the Solarbio PC0010 Kit (Li et al., 2019). NH3-N levels were measured colorimetrically according to our established analytical protocol (Wang et al., 2021).

### 2.4 Metagenomic binning

The gene abundance of the *Prevotella* genus was investigated using a metagenomic assembly and binning approach. To maximize the recovery of assembled contigs, both individual assemblies for each time point sample and co-assemblies of high-quality reads from porcine colon samples were performed using MEGAHIT (v1.1.1; parameters: --min-contig-len 500 -t 40) (Li et al., 2015, 2016). Contigs (>1.5 kb) from single-sample assemblies and co-assemblies were independently subjected to metagenomic binning based on sequence composition and coverage depth using three tools with default parameters: MaxBin (v2.2.4) (Wu et al., 2016; Wu and Singer, 2021), MetaBAT2 (v2.11.1) (Kang et al., 2015), and CONCOCT (v0.4.0) (Alneberg et al., 2014). The resulting metagenome-assembled genomes (MAGs) were consolidated using DAS Tool (v1.1.1) (Sieber et al., 2018). High-quality MAGs (completeness >90%, contamination <5%) were identified using CheckM (v1.0.7; lineage_wf workflow) (Parks et al., 2015). Dereplication at 99% average nucleotide identity (ANI) was performed with dRep (v2.5.4; parameters: -p 72 --ignore_genome_quality -pa 0.95 -sa 0.99 -cm larger) (Olm et al., 2017).

### 2.5 Genome annotation

The complete genome sequences of *Prevotella copri* (accession number: CP085932.1) and *Prevotella* sp. *MGM2* (accession number: NZ_BEWW00000000.1) were retrieved from the National Center for Biotechnology Information (NCBI). To comprehensively investigate gene functions of these two *Prevotella* species, all coding genes underwent systematic functional annotation using the Clusters of Orthologous Groups (COG) database^2^ and the Kyoto Encyclopedia of Genes and Genomes (KEGG) database^3^, where COG focuses on protein functional classification and KEGG emphasizes metabolic pathway annotation.

### 2.6 Statistical analyses and visualization

Daily fluctuations of Bacteroidota and *Prevotella* were detected using JTK_cycle (Hughes et al., 2010) over a 24-h period at 3-h intervals. *P*_Adj_ < 0.05 was considered indicative of significant diurnal oscillation; only microbial taxa with a relative abundance >0.01% and present in >20% of samples were retained for further analysis (Samad et al., 2022). Correlations among *Prevotella*, colonic nutrient substrates, KEGG genes, contributing taxa, and KEGG pathways were calculated using Extended Local Similarity Analysis (eLSA) (Gálvez et al., 2020). Local Similarity (LS) coefficients were measured pairwise to quantify correlation levels between variables. The delay value (D) represented the time shift between pairwise comparisons.

Temporal similarity was assessed via constrained Principal Coordinate Analysis (cPCoA) using ImageGP^4^. Visualizations were generated as follows: Pie charts, line graphs, stacked columns, and scatter plots of rhythmic species were generated using Origin 2024. Heatmaps were generated with TBtools-II (v2.154). Network diagrams were plotted using Gephi (v0.10.1).

## 3 Results

### 3.1 General overview of Bacteroidota in the colon

**Figure 1:**
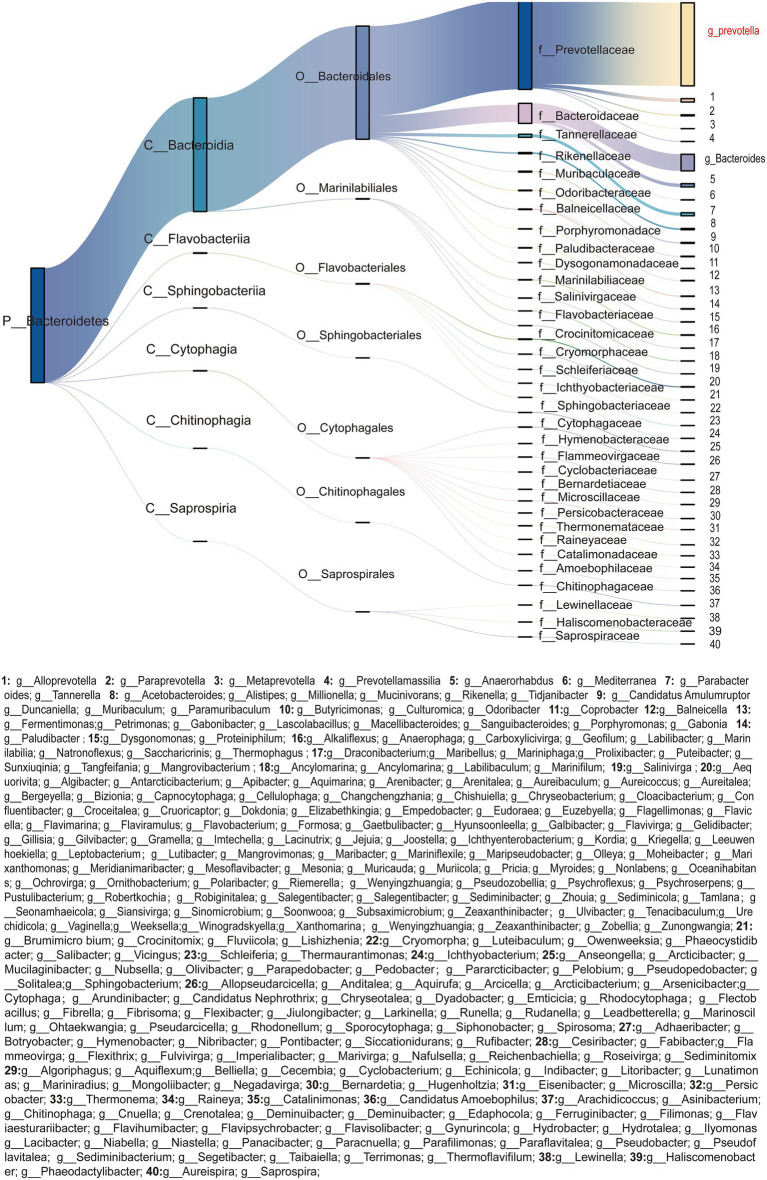
Phylogenetic composition and hierarchical flux of Bacteroidota (*n* = 5) taxa in the porcine colon. Sankey diagram illustrating the taxonomic composition of Bacteroidota (*n* = 5) across five hierarchical ranks (Phylum, Class, Order, Family, Genus). Columns represent distinct taxonomic ranks, with node widths proportional to their relative abundances. Streamlines denote the flow of microbial biomass between ranks, Line thickness correlates with taxon-specific contributions to community structure (Bray–Curtis similarity > 0.75, *P*_Adj_ < 0.05). Nodes are color-coded by phylogenetic affiliation, with insets emphasizing key genus [*Prevotella* (*n* = 5) and *Bacteroides* (*n* = 5)].

In the colonic ecosystem, Bacteroidota occupy the core ecological niche, with Bacteroidales as the dominant lineage. Among these, the predominant bacterial family was Prevotellaceae followed by Bacteroidaceae (Figure 1). At the genus level, the relative abundance of *Prevotella* significantly surpassed that of other genera, demonstrating clear ecological dominance. Additionally, *Bacteroides* exhibited relatively notable ecological advantages as a secondary dominant genus.

### 3.2 Composition and fluctuation of phylum Bacteroidota and genus Prevotella in the colon of growing pigs

**Figure 2:**
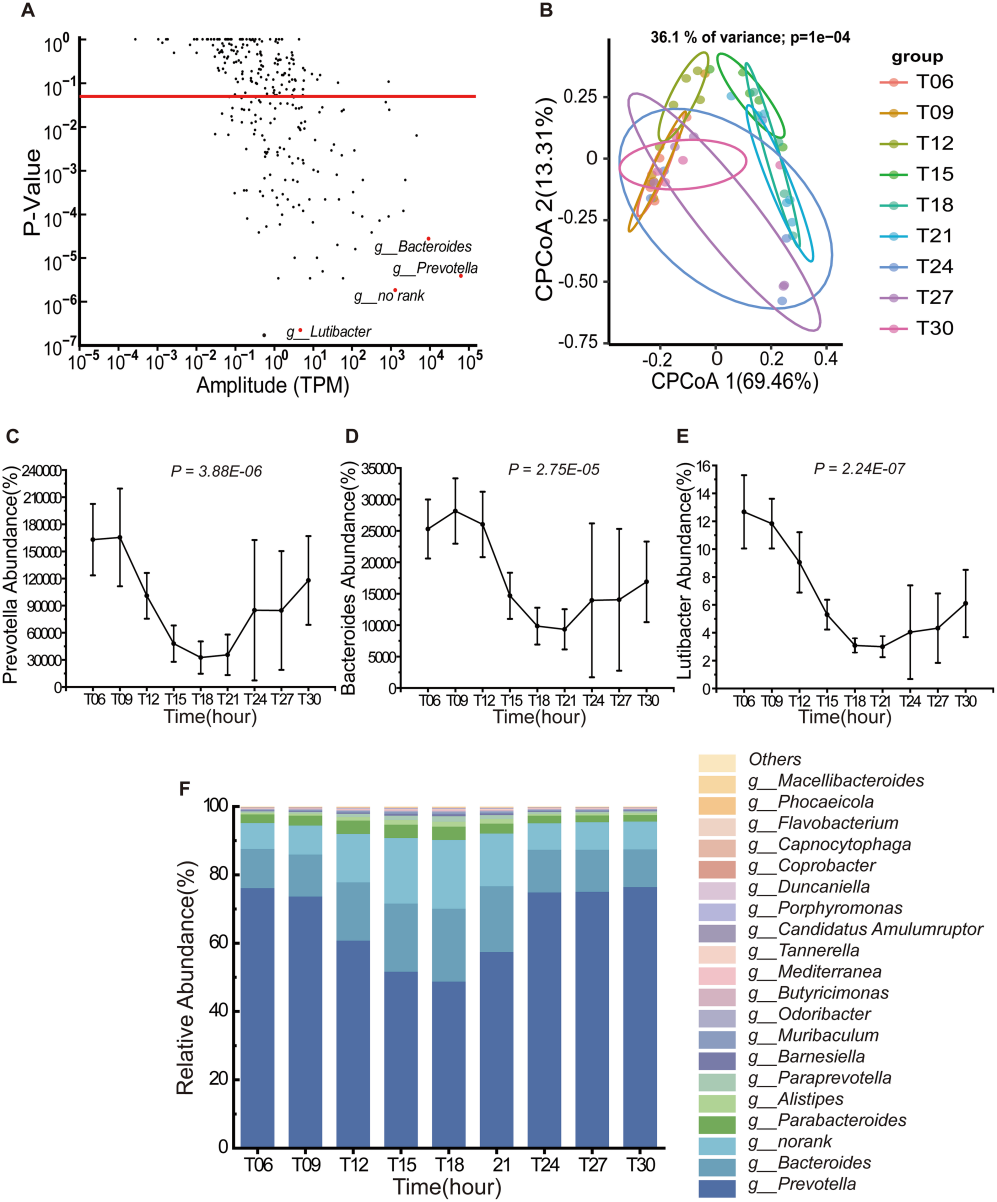
Temporal dynamics of Bacteroidota (*n* = 5) genera in the porcine colon. **(A)** Diurnal oscillations of bacterial genera within the phylum Bacteroidota (*n* = 5) over a 24-h period. The red dashed line denotes the threshold for statistical significance (*P*_Adj_ < 0.05). **(B)** Constrained Principal Coordinates Analysis (CPCoA) of Bray–Curtis dissimilarity indices, stratified by Bacteroidota (*n* = 5) (36.1% variance explained; PERMANOVA, *p* = 0.0001). Samples are color-coded by different time point, with distinct shapes denoting sampling intervals (T06-T21). Axes labels indicate the proportion of total variance captured by each principal coordinate. **(C–E)** Rhythmic fluctuations of three representative genera [*Prevotella* (*n* = 5), *Bacteroides* (*n* = 5) and *Lutibacter* (*n* = 5)] across a daily cycle. Data represent mean ± SEM (*n* = 5 biological replicates). **(F)** Hierarchical abundance profile of the top 20 Bacteroidota (*n* = 5) genera in the colonic ecosystem.

At the genus level, 36.73% of taxa exhibited statistically significant oscillations (*P*_Adj_ < 0.05), with *Prevotella*, *Bacteroides* and *Lutibacter* displaying pronounced rhythmicity (Figure 2A). Within the Bacteroidota phylum, *Prevotella*, *Bacteroides*, and *Lutibacter* exhibited conserved diurnal patterns, peaking between T06-T09 and declining to minimal levels from T18 to T21 (Figures 2C–E). Among the top 20 genera by relative abundance, *Prevotella* and *Bacteroides* collectively dominated the microbial landscape, accounting for 83.81% of the total abundance (Figure 2F). Notably, *Prevotella* maintained the highest relative abundance across all time points, followed by *Bacteroides*. cPCoA based on Bray–Curtis dissimilarity revealed significant temporal structuring of microbial communities (36.1% variance explained; PERMANOVA, *p* = 0.0001, Figure 2B). Samples clustered into two temporal clusters: T15, T18, and T21 formed a distinct nocturnal cluster, while T06, T09, and T12 comprised a diurnal cohort.

**Figure 3:**
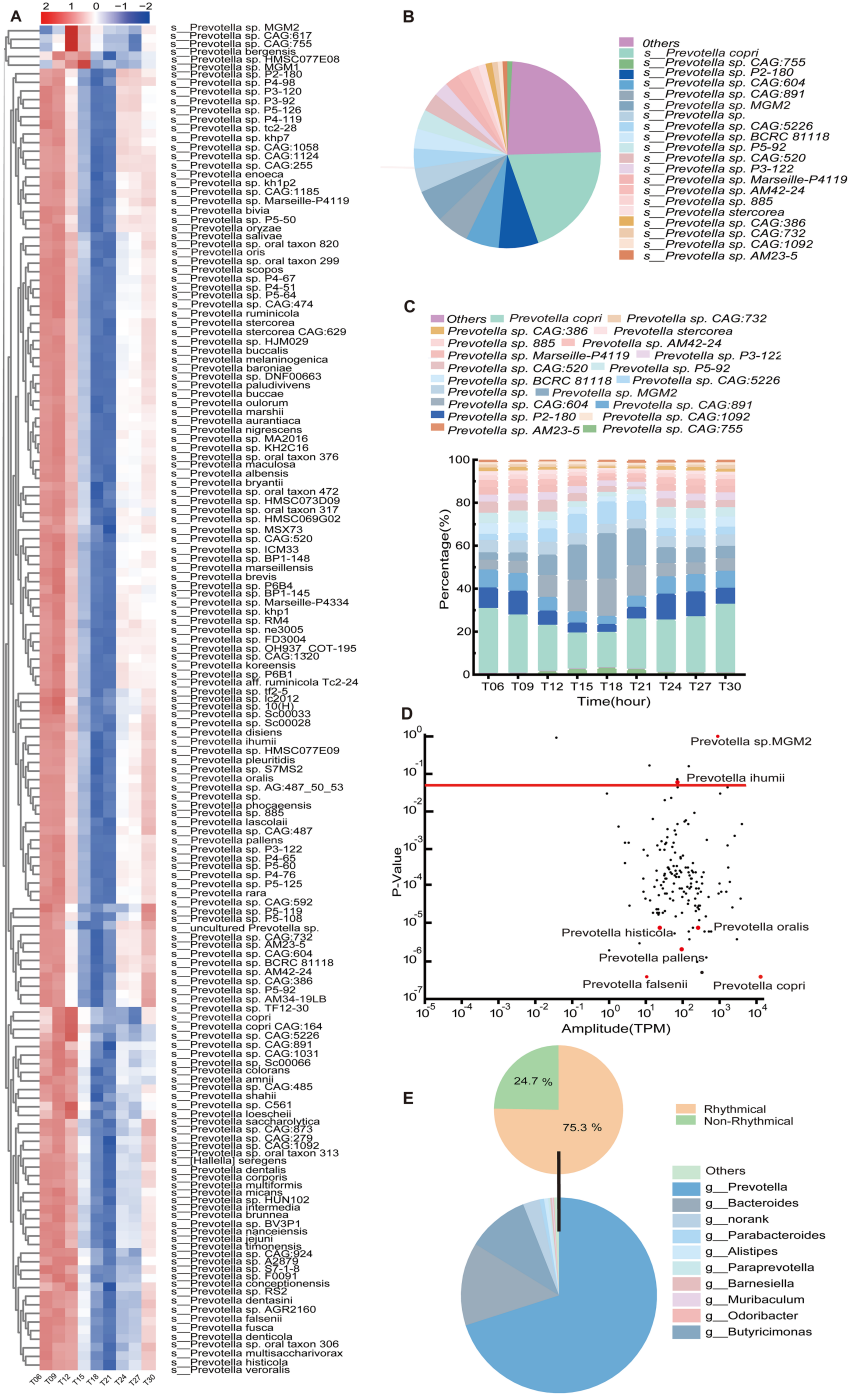
Temporal dynamics and ecological hierarchy of *Prevotella* (*n* = 5) in the porcine colon. **(A)** Heatmap of Z-score-normalized relative abundance of *Prevotella* (*n* = 5) species at 9 time points. Row clustering reflects time-dependent aggregation patterns, with row annotations indicating class-level phylogenetic classification. **(B)** Rank-abundance curve of the top 20 *Prevotella* (n = 5) species, highlighting *Prevotella copri* (*n* = 5) dominance. **(C)** Composition and fluctuations of the key gut microbial taxa *Prevotella* (*n* = 5) genus (top 20) at different time points. **(D)** Diurnal trajectories of *Prevotella* (*n* = 5) species abundance, with the red dashed line marking the significance threshold (*P*_Adj_ < 0.05). **(E)** Upper panel: Proportional distribution of rhythmic (*P*_Adj_ < 0.05) versus non-rhythmic taxa within the phylum Bacteroidota (*n* = 5). Lower panel: Ranked relative abundance of the top 10 genera in Bacteroidota (*n* = 5), ordered by daily oscillation amplitude.

At the species level, *Prevotella copri* dominated among the *Prevotella* species, representing 20.04% of the total microbial abundance (Figure 3B). Temporally resolved heatmap analysis demonstrated diurnal fluctuations in 156 *Prevotella* species across nine time points (Figure 3A). These species exhibited conserved biphasic dynamics: relative abundance peaked during early light-phase intervals (T06 and T09, corresponding to postprandial periods) and declined sharply at T18 and T21 (dark-phase troughs), reaching minimal levels at T18 (Figure 3C). Daily fluctuation analysis via the JTK_Cycle algorithm identified 150 rhythmic *Prevotella* species (*P*_Adj_ < 0.05). Notably, *Prevotella copri* exhibited strong amplitude and daily rhythmic fluctuations, whereas *Prevotella* sp. *MGM2* displayed high-amplitude fluctuations without daily fluctuations (*P*_Adj_ > 0.05) (Figure 3D). Within the Bacteroidota, 75.3% of genera exhibited statistically significant daily oscillations (*P*_Adj_ < 0.05), with *Prevotella* and *Bacteroides* dominating this rhythmic hierarchy. Strikingly, *Prevotella* emerged as the predominant genus in the porcine colon, constituting 70.04% of the total Bacteroidota population (Figure 3E).

### 3.3 Dynamic *Prevotella*-colonic nutrient substrate interactions

**Figure 4:**
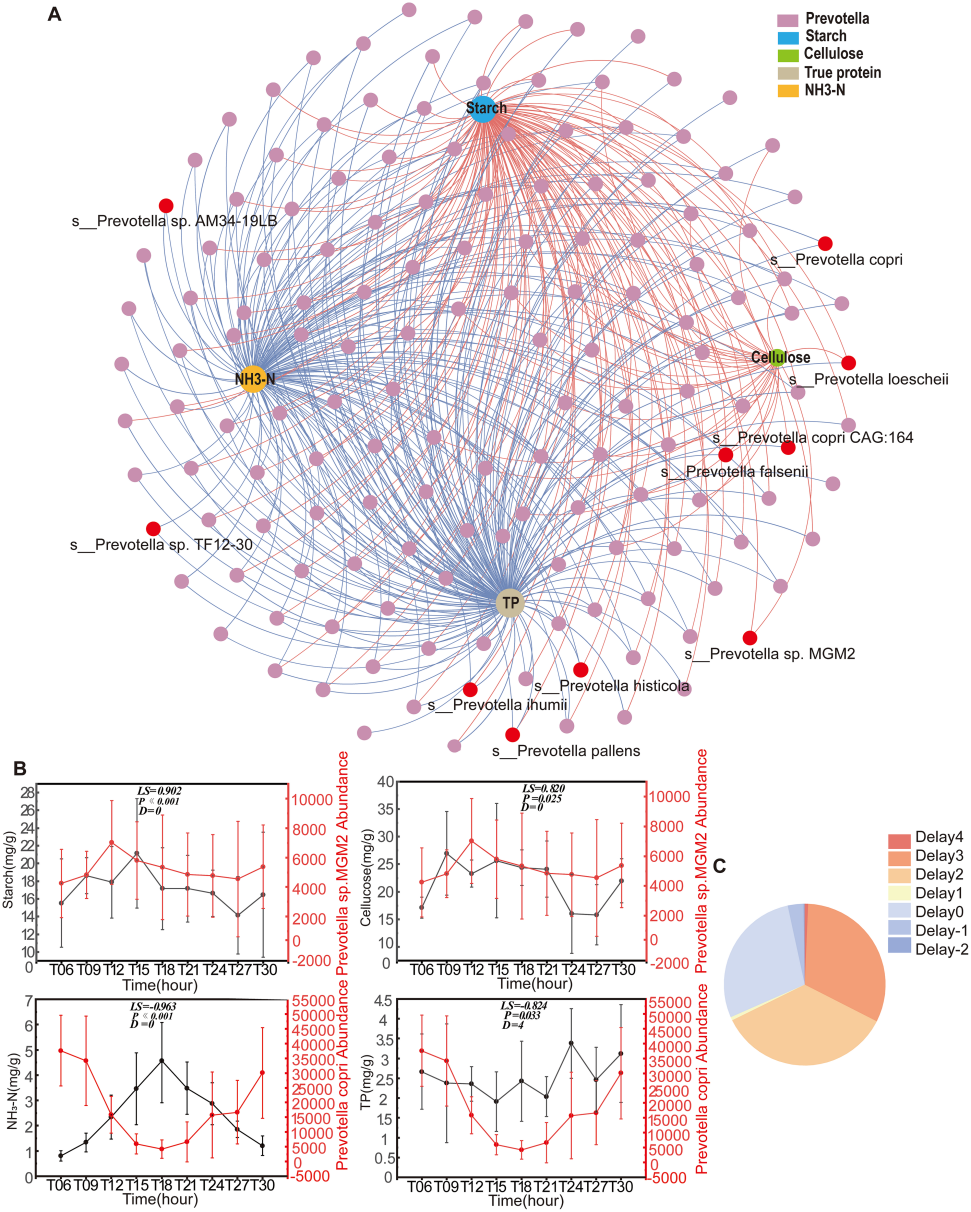
Nutrient-*Prevotella* interaction network and temporal coupling dynamics. **(A)** The network analysis revealing the relationship between nutrient substrates and *Prevotella* (*n* = 5) genus. The nodes are colored according to the different nutrient substrates and *Prevotella* (*n* = 5) genus. The network connection represents significant (*P*_Adj_ < 0.05) correlation. The size of each node is proportional to the number of connections. **(B)** Diurnal fluctuations of nutrient substrates and representative *Prevotella* (*n* = 5) species over the course of a day. The data are presented as the mean ± SEM. The left vertical axis represents the abundance of nutrient substrates, and the right vertical axis represents the abundance of *Prevotella* (*n* = 5) species. **(C)** Pie chart showing the percentage of the correlation pairwise with different time shifts. Pairwise correlation with a negative delay means the metabolism of nutrient substrates that the was ahead of the *Prevotella* (*n* = 5) genus, whereas pairwise correlation with a positive time delay means that the *Prevotella* (*n* = 5) genus was ahead of the metabolism of nutrient substrates.

A co-occurrence network integrating *Prevotella* and colonic nutrient substrates revealed potential ecological interactions, comprising 160 nodes (156 *Prevotella* species and 4 substrates: TP, NH3-N, starch, and cellulose) with 443 edges (Figure 4A). Network topology analysis identified contrasting substrate associations: *Prevotella* exhibited negative correlations with TP and NH3-N while demonstrating strong positive correlations with starch and cellulose. Time-lagged cross-correlation analysis via the eLSA algorithm, which accounted for temporal offsets of −2 to 4 h, elucidated bidirectional substrate-microbe dynamics. Notably, 64.56% of interactions displayed negative time delays, suggesting that nutrient availability drives *Prevotella* population dynamics. Conversely, 35.44% exhibited positive time delays, implicating microbial enzymatic activity in substrate degradation (Figure 4C). *Prevotella* sp. *MGM2* exhibited positive correlations with starch and cellulose without a time delay, while *Prevotella copri* showed negative correlations with NH_3_-N and TP, with a 4-h delay in its interaction with TP (Figure 4B).

### 3.4 Functional differences between rhythmic and non-rhythmic species

**Figure 5:**
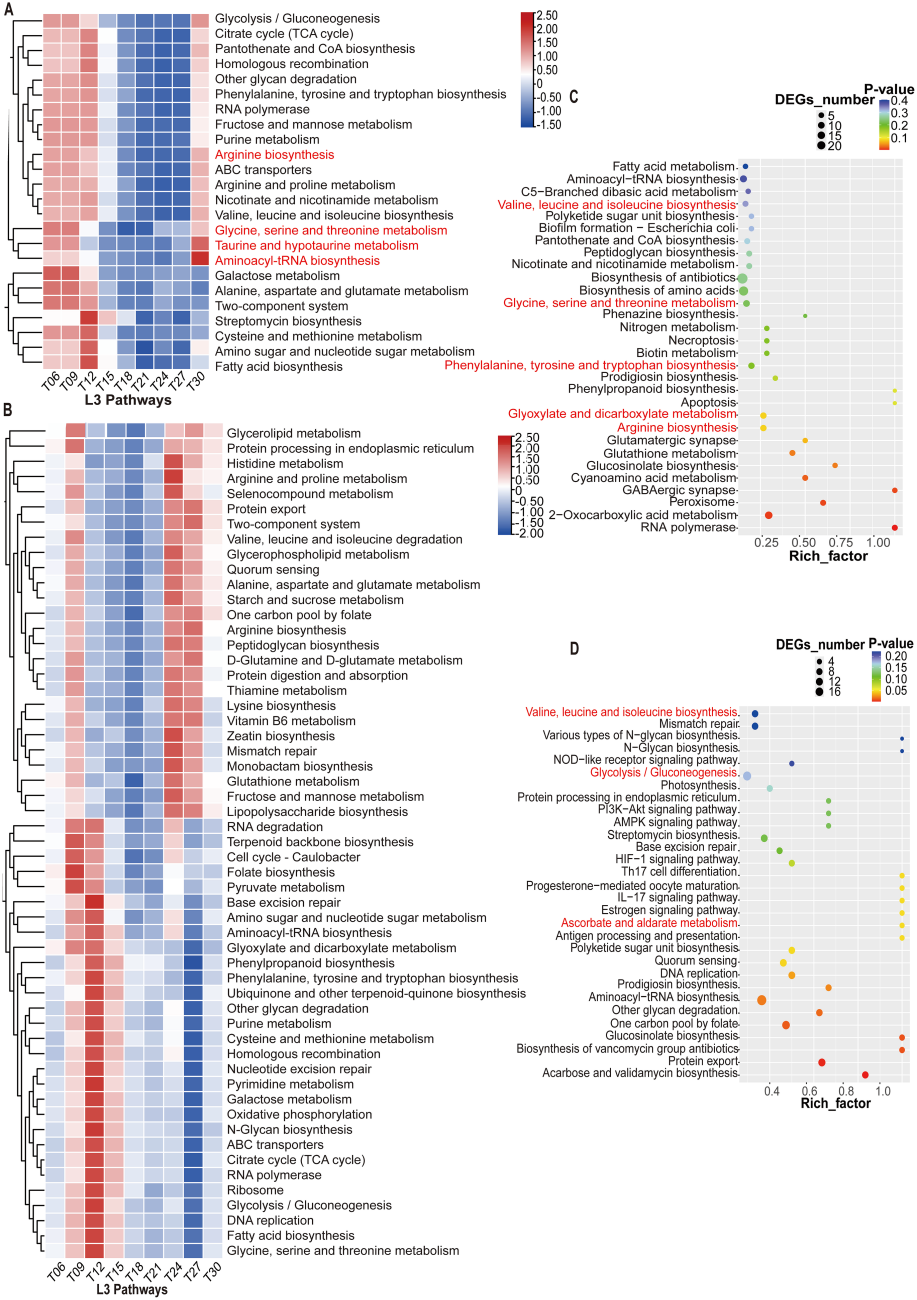
Heatmap of relative abundance of KEGG Level-3 metabolic pathways in two *Prevotella* (*n* = 5) species across nine time points. **(A)** The relative abundances of KEGG level-3 metabolic pathways of *Prevotella copri* (*n* = 5) across different time points. **(B)** The relative abundances of KEGG level-3 metabolic pathways of *Prevotella* sp. *MGM2* (*n* = 5) across different time points **(C)** KEGG pathway enrichment bubble plot of *Prevotella copri* (*n* = 5). X-axis: Rich factor, representing the ratio of the number of differentially expressed genes to the total annotated genes in the pathway; Y-axis: Significantly enriched KEGG pathway names (sorted in descending order of Rich factor); Bubble size: Number of genes enriched in the pathway; Bubble color: *p*-value, with deeper red indicating higher enrichment significance. **(D)** KEGG pathway enrichment bubble plot of *Prevotella* sp. *MGM2* (*n* = 5).

Through KEGG pathway enrichment analysis of gene IDs identified from two *Prevotella* species in the colon, we identified metabolic or signaling pathways significantly enriched in these genes. In *Prevotella copri*, significantly enriched carbohydrate metabolism included glyoxylate and dicarboxylate metabolism, while amino acid metabolism were primarily phenylalanine, tyrosine and tryptophan biosynthesis; valine, leucine and isoleucine biosynthesis; glycine, serine and threonine metabolism; and arginine biosynthesis (Figure 5C). In *Prevotella* sp. *MGM2*, significantly enriched carbohydrate metabolism included ascorbate and aldarate metabolism and glycolysis/gluconeogenesis, whereas amino acid metabolism were dominated by valine, leucine and isoleucine biosynthesis (Figure 5D).

As the *Prevotella* with the most pronounced daily fluctuation and highest relative abundance in the genus, *Prevotella copri* was selected as the rhythmic signature species. In contrast, *Prevotella* sp. *MGM2*, which exhibited the highest relative abundance among non-rhythmic species and weakest rhythmicity, served as the representative non-rhythmic species. Following metagenomic sequencing data assembly and binning, metagenome-assembled genomes (MAGs) were taxonomically classified at the species level. Subsequent analysis focused on annotated KEGG metabolic pathways for both species. The relative abundances of KEGG level-3 metabolic pathways of *Prevotella copri* showed significant temporal fluctuations, with glycine, serine and threonine metabolism; taurine and hypotaurine metabolism; arginine biosynthesis; and aminoacyl-tRNA biosynthesis demonstrating statistically significant rhythmic variations (*P*_Adj_ < 0.05) (Figure 5A). Conversely, *Prevotella* sp. *MGM2* displayed no consistent temporal trends in its KEGG level-3 pathways. A subset of pathways exhibited increased relative abundance from T09 to T15, reaching minima at T27, while another subset peaked at T09 and T24-T27 (Figure 5B).

**Figure 6:**
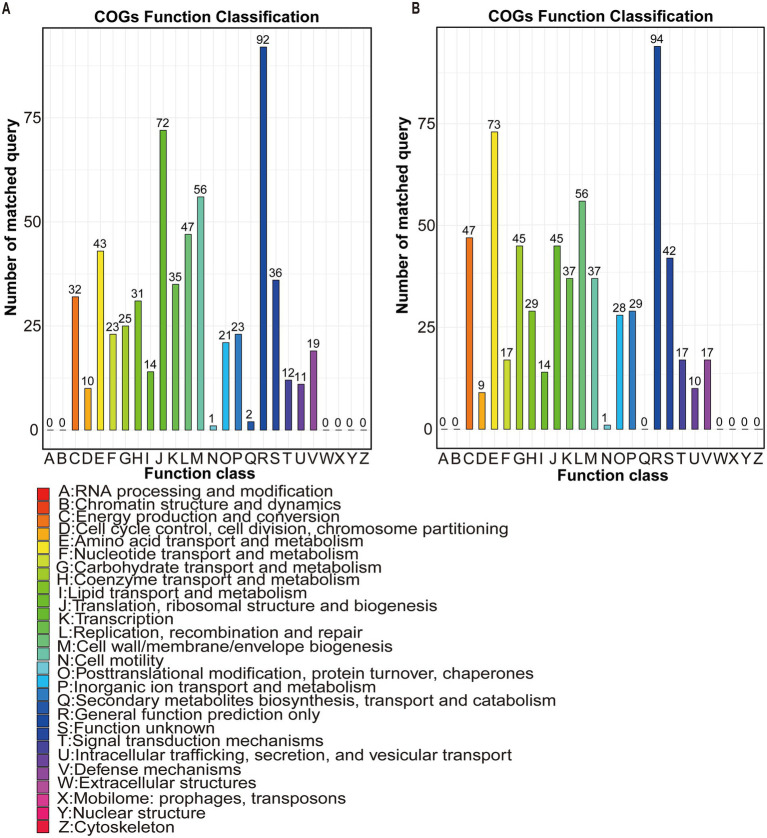
Comparative functional annotation of *Prevotella copri* and *Prevotella* sp. *MGM2*: Integrated KEGG pathway enrichment and COG category profiling reveal strain-specific metabolic divergence. **(A)** COG functional annotation profile of *Prevotella copri*. Color: Represents COG functional categories; Bar height: Number of differentially expressed genes annotated to each functional category; X-axis: COG functional categories (sorted in descending order of gene count); Y-axis: Number of annotated genes; (COG classification is based on the NCBI database with BLASTp alignment E-value ≤ 1e-5^2, 5^). **(B)** COG functional annotation profile of *Prevotella* sp. *MGM2*.

This study retrieved the complete genome sequences of both species from the National Center for Biotechnology Information (NCBI) for functional analyses. COG annotation revealed distinct metabolic profiles. *Prevotella* sp. *MGM2* possessed 45 genes in carbohydrate transport and metabolism (G) and 73 genes in amino acid transport and metabolism (E), while *Prevotella copri* contained 31 and 43 genes in these respective COG categories, demonstrating significantly higher G/E category gene counts in *Prevotella* sp. *MGM2* (Figures 6A,B).

### 3.5 Gene-level correlations between rhythmic and non-rhythmic bacteria and nutrient substrates

**Figure 7:**
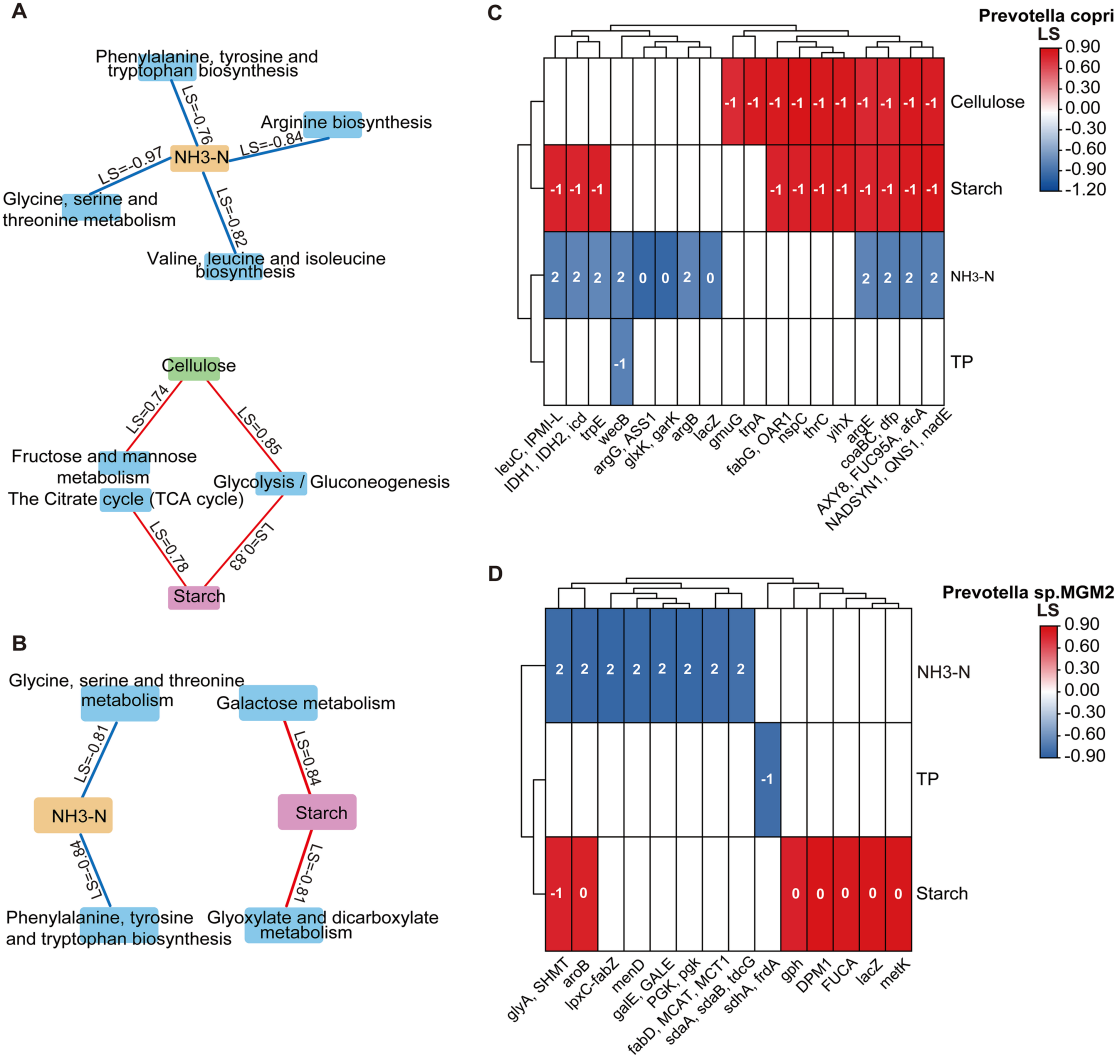
Dynamic correlation network between colonic nutrient substrates and KEGG Level-3 metabolic pathways/gene interactions. **(A)** Network diagram of *Prevotella copri* KEGG level-3 metabolic pathways and their associations with nutrient substrates. **(B)** Network diagram of *Prevotella* sp. *MGM2* KEGG level-3 metabolic pathways and their associations with nutrient substrates. Red lines indicate positive correlations, and blue lines indicate negative correlations, with *P*_Adj_ representing the LS values between them. **(C)** Dynamic correlation plot between genes and nutrient substrates in *Prevotella copri* (*n* = 5); the number within each cell represents the time delay between them. **(D)** Dynamic correlation plot between genes and nutrient substrates in *Prevotella* sp. *MGM2* (*n* = 5); the number within each cell represents the time delay between them.

Based on the dynamic correlation model eLSA, general relationships were established between KEGG level-3 metabolic pathways, genes, and nutrient substrates for two *Prevotella* species. In *Prevotella copri*, Based on the dynamic correlation model eLSA, dynamic relationships were established between KEGG level-3 metabolic pathways, genes, and nutrient substrates for two Prevotella species. In *Prevotella copri*, three carbohydrate metabolism-related level-3 pathways were identified: the citrate cycle (TCA cycle) and glycolysis/gluconeogenesis showed positive correlations with starch, while fructose and mannose metabolism and glycolysis/gluconeogenesis exhibited positive correlations with cellulose. Additionally, four amino acid metabolism-related pathways—arginine biosynthesis; glycine, serine and threonine metabolism; valine, leucine and isoleucine biosynthesis; and phenylalanine, tyrosine and tryptophan biosynthesis—all showed negative correlations with NH₃-N (Figure 7A). In *Prevotella* sp. *MGM2*, carbohydrate metabolism pathways including galactose metabolism and glyoxylate and dicarboxylate metabolism were positively correlated with starch, while in amino acid metabolism, glycine, serine and threonine metabolism and phenylalanine, tyrosine and tryptophan biosynthesis showed negative correlations with NH_3_-N (Figure 7B).

Genes from both species showed positive correlations with starch and cellulose and negative correlations with NH₃-N and TP, with all interactions exhibiting time delays ranging from −1 to 2 h. In *Prevotella copri*, carbohydrate metabolism-related genes *IDH1* (LS = 0.78), *IDH2* (LS = 0.78), *icd* (LS = 0.78), and *yihX* (LS = 0.83) correlated positively with starch (delay = −1), while *gmuG* (LS = 0.74) and *yihX* (LS = 0.85) showed positive correlations with cellulose (delay = −1). Amino acid metabolism-related genes *argB* (LS = −0.78), *argE* (LS = −0.78), *argG* (LS = −0.97), *ASS1* (LS = −0.97), *glxK* (LS = −0.97), *garK* (LS = −0.97), *trpE* (LS = −0.76), *leuC* (LS = −0.82), and *IPMI-L* (LS = −0.82) exhibited negative correlations with NH_3_-N (delay = 0 or 2) (Figure 7C). In *Prevotella* sp. *MGM*2, carbohydrate metabolism-related gene *gph* (LS = 0.78) correlated positively with starch (delay = 0). Amino acid metabolism-related genes *glyA* (LS = −0.82), *SHMT* (LS = −0.82), *sdaA* (LS = −0.79), *sdaB* (LS = −0.79), *tdcG* (LS = −0.79), and *aroB* (LS = −0.84) showed negative correlations with NH_3_-N (delay = 2) (Figure 7D). Additionally, four amino acid metabolism-related pathways—arginine biosynthesis, glycine, serine and threonine metabolism, valine, leucine and isoleucine biosynthesis, and phenylalanine, tyrosine and tryptophan biosynthesis—all showed negative correlations with NH_3_-N (Figure 7A). In *Prevotella* sp. *MGM2*, carbohydrate metabolism pathways including galactose metabolism and glyoxylate and dicarboxylate metabolism were positively correlated with starch, while in amino acid metabolism, glycine, serine and threonine metabolism and phenylalanine, tyrosine and tryptophan biosynthesis showed negative correlations with NH_3_-N (Figure 7B).

Genes from both species showed positive correlations with starch and cellulose and negative correlations with NH_3_-N and TP, with time delays ranging from −1 to 2 h. In *Prevotella copri*, carbohydrate metabolism-related genes *IDH1* (LS = 0.78), *IDH2* (LS = 0.78), *icd* (LS = 0.78), and *yihX* (LS = 0.83) correlated positively with starch (delay = −1), while *gmuG* (LS = 0.74) and *yihX* (LS = 0.85) showed positive correlations with cellulose (delay = −1). Amino acid metabolism-related genes *argB* (LS = −0.78), *argE* (LS = −0.78), *argG* (LS = −0.97), *ASS1* (LS = −0.97), *glxK* (LS = −0.97), *garK* (LS = −0.97), *trpE* (LS = −0.76), *leuC* (LS = −0.82), and *IPMI-L* (LS = −0.82) exhibited negative correlations with NH3-N (delay = 0 or 2) (Figure 7C). In *Prevotella* sp. *MGM2*, carbohydrate metabolism-related gene *gph* (LS = 0.78) correlated positively with starch (delay = 0). Amino acid metabolism-related genes *glyA* (LS = −0.82), *SHMT* (LS = −0.82), *sdaA* (LS = −0.79), *sdaB* (LS = −0.79), *tdcG* (LS = −0.79), and *aroB* (LS = −0.84) showed negative correlations with NH3-N (delay = 2) (Figure 7D).

## 4 Discussion

Previous studies in porcine models have demonstrated that fluctuations in colonic microbiota are closely associated with oscillations in nutrient substrates (Wang et al., 2023), but the dynamic correlations between specific bacterial species and nutrient substrates remain unclear. This study focused on *Prevotella*, which exhibits significant daily fluctuations, to investigate its dynamic interactions with colonic nutrient substrates and elucidate the genetic basis underlying its daily fluctuations. As a dominant genus within the Bacteroidota, *Prevotella* serves as a key dietary-fiber-fermenting bacterium that degrades complex polysaccharides (e.g., plant fibers) to generate short-chain fatty acids (SCFAs) such as acetate, propionate, and butyrate, which provide energy for intestinal epithelial cells and maintain barrier integrity (Kovatcheva Datchary et al., 2015; Gálvez et al., 2020).

This study employed a fistulated pig model to enable continuous sampling within the same individual, with the aim of reducing inter-individual variability. However, under the current experimental conditions, we acknowledge that while the fistulated model offers practical advantages (e.g., enabling long-term repeated non-invasive sampling with reduced animal stress compared to terminal methods), surgical modification may alter local microenvironmental factors (e.g., luminal flow or mucosal barrier function). Consequently, minor discrepancies between sampling data and the true physiological state (e.g., fluctuations in feed intake) may persist. Colonic nutrient substrate content showed high synchronization with feeding rhythms, and microbial abundance fluctuations were strongly correlated with nutrient substrate variations. Previous studies confirmed that in murine models, Bacteroidota and Verrucomicrobia peak during fasting periods, while Firmicutes dominate during feeding periods (Zarrinpar et al., 2014). Human studies revealed diurnal oscillations in 15.2% of microbial operational taxonomic units (OTUs), with Bacteroidota peaking nocturnally and Firmicutes diurnally, accompanied by cyclic fluctuations in alpha-diversity (Reitmeier et al., 2020). Our findings demonstrated that Bacteroidota abundance peaked at T06-T09 and reached trough levels at T18-T21. Within this phylum, 75.3% of species exhibited daily fluctuations, among which *Prevotella* showed the highest relative abundance and most pronounced rhythmic patterns. Notably, dynamic correlation analysis between *Prevotella* and colonic nutrient substrates revealed negative correlations with NH_3_-N (ammonia nitrogen)/TP (total phosphorus) and positive correlations with starch/cellulose, potentially attributable to *Prevotella*’s metabolic capability to degrade host-indigestible complex fibers for energy acquisition, thereby promoting microbial proliferation.

Further analysis of different species within *Prevotella* revealed that *Prevotella copri* exhibits the highest relative abundance and most pronounced daily fluctuations. Current studies demonstrate its dual roles: improving insulin sensitivity and lipid metabolism (Gong et al., 2024; Ismail et al., 2025). Murine experiments indicate that oral administration of *Prevotella copri* alleviates traumatic brain injury (TBI)-induced neurological deficits (motor/cognitive dysfunction), anxiety-like behaviors, oxidative stress, blood–brain barrier damage, and neuronal apoptosis (Gu et al., 2024), suggesting its probiotic potential. However, *Prevotella copri* exacerbates vascular calcification in chronic kidney disease via LPS-mediated gut barrier disruption and NF-κB/NLRP3 inflammasome activation (Hao et al., 2024); meanwhile, it depletes tryptophan (Trp) in breast cancer patients to reduce anticancer indole-3-pyruvate (IPyA) levels and promote tumor progression (Su et al., 2024); furthermore, it correlates with elevated lipopolysaccharide (LPS) levels under high-fat diets, aggravating obesity and systemic inflammation (Yuan et al., 2024; Ismail et al., 2025); and shows no significant colonization changes under high-fiber diets but inhibits fat accumulation by reducing energy intake (Chen et al., 2021). It thrives competitively through the polysaccharide utilization loci (PULs) that efficiently metabolize complex xylans, influencing host health via dietary fiber metabolism (Linares-Pastén et al., 2021). *Prevotella copri* comprises four genetically distinct clades (Clade A-D) (Tett et al., 2019), with clade-specific metabolic functions (Iljazovic et al., 2021), which may explain the bidirectional effects of *prevotella copri* in disease contexts.

*Prevotella copri* demonstrates higher abundance in the gut microbiota of non-Westernized populations (consuming plant-based diets) and harbors carbohydrate-active enzymes (CAZymes) that interact with high-fiber diets to improve glucose homeostasis (Jiang et al., 2022), whereas its abundance is reduced under Westernized diets (high-fat, high-protein) (Tett et al., 2019). Our findings reveal that both *Prevotella copri* and *Prevotella* sp. *MGM2* show significant enrichment in KEGG pathways related to amino acid metabolism, carbohydrate metabolism, lipid metabolism, and biosynthesis of secondary metabolites. These pathways may mediate host–microbe interactions through amino acid fermentation (e.g., SCFA production), carbohydrate breakdown (e.g., dietary fiber utilization), and lipid metabolism, thereby influencing inflammation and energy homeostasis. Secondary metabolites (e.g., vitamins/neurotransmitter precursors) may further modulate host physiology via the gut-brain axis (Ogata et al., 1999; Kanehisa et al., 2017, 2023). Notably, *Prevotella copri* exhibited statistically significant rhythmic variations (*p* < 0.05) in metabolic pathways including glycine/serine/threonine metabolism, taurine and hypotaurine metabolism, arginine biosynthesis, and aminoacyl-tRNA biosynthesis. In contrast, *Prevotella* sp. *MGM2* displayed no consistent temporal trends in the relative abundance of its KEGG level-3 pathways.

Comparative analysis of core gene functions in *Prevotella copri* and *Prevotella* sp. *MGM2* based on the COG database revealed high gene abundance in functional categories Carbohydrate Transport and Metabolism (G) and Amino Acid Transport and Metabolism (E) for both species, where enrichment of category G genes suggests genetic potential to degrade host-indigestible complex polysaccharides (e.g., cellulose, starch) or utilize diverse monosaccharides (e.g., glucose, fructose), which confers ecological advantages, while category E gene abundance indicates efficient amino acid uptake and metabolism (Tatusov, 2000); notably, *Prevotella* sp. *MGM2* harbored significantly higher gene counts in G (45 genes) and E (73 genes) than *Prevotella copri* (31 genes in G; 43 genes in E), yet demonstrated fewer nutrient substrates-related KEGG level-3 pathways and annotated genes—specifically, *Prevotella* sp. *MGM2* had only two KEGG level-3 pathways (galactose metabolism and glyoxylate and dicarboxylate metabolism) and one gene (*gph*) linked to starch, while two pathways (glycine, serine and threonine metabolism and phenylalanine, tyrosine and tryptophan biosynthesis) and six genes (*glyA*, *SHMT*, *sdaA*, *sdaB*, *tdcG*, *aroB*) were associated with NH₃-N; in contrast, *Prevotella copri* exhibited two pathways (citrate cycle [TCA cycle] and glycolysis/gluconeogenesis) and four genes (*IDH1*, *IDH2*, *icd*, *yihX*) correlated with starch, two pathways (fructose and mannose metabolism and glycolysis/gluconeogenesis) and two genes (*gmuG*, *yihX*) linked to cellulose, and four pathways (arginine biosynthesis, glycine/serine/threonine metabolism, valine/leucine/isoleucine biosynthesis, and phenylalanine/tyrosine/tryptophan biosynthesis) and nine genes (*argB*, *argE*, *argG*, *ASS1*, *glxK*, *garK*, *trpE*, *leuC*, *IPMI-L*) associated with NH₃-N.

These findings highlight that *Prevotella* sp. *MGM2* possesses fewer nutrient-substrates-related KEGG level-3 pathways and functional genes than *Prevotella copri*. Importantly, the relative abundances of *Prevotella copri*’s KEGG level-3 pathways exhibited dynamic fluctuations synchronized with colonic substrate concentration variations and host feeding rhythms, whereas *Prevotella* sp. *MGM2*’s pathway abundances lacked consistent temporal oscillations. This lack of temporal oscillations suggests that *Prevotella* sp. *MGM2*’s metabolic activity is less influenced by substrate concentration fluctuations and exhibits time-independent regulation of pathway activity. Conversely, *Prevotella copri* likely employs a rhythmic substrate-responsive metabolism, aligning its metabolic efficiency with spatiotemporal variations in substrate availability.

## 5 Conclusion

In summary, this study investigated the daily fluctuations of the *Prevotella* and its interaction with colonic nutrient substrates, revealing distinct correlation patterns: NH_3_-N and TP exhibited significant negative correlations with *Prevotella*, whereas cellulose and starch showed strong positive correlations. Notably, *Prevotella copri* displayed the highest abundance and most robust daily fluctuations, whereas *Prevotella* sp. *MGM2* had relatively high abundance but lacked daily fluctuations. Comparative analysis demonstrated that *Prevotella* sp. *MGM2* harbored higher gene abundances in COG categories Carbohydrate Transport and Metabolism and Amino Acid Transport and Metabolism, yet possessed fewer nutrient-substrates-related KEGG level-3 pathways and functionally annotated genes than *Prevotella copri*. These findings suggest that *Prevotella* species with different daily fluctuations may adopt different response strategies to nutrient substrates: *Prevotella copri* may employ a rhythmic substrate-responsive strategy, synchronizing its relative abundance, metabolic activity, and gene expression with temporal variations in colonic substrate concentrations; in contrast, *Prevotella* sp. *MGM2* may adopt a sustained response strategy, characterized by stable metabolic activity independent of fluctuations in substrate concentrations.

## Data Availability

The datasets presented in this study are publicly available. This data can be found here: https://www.ncbi.nlm.nih.gov, accession number PRJNA843783.

## Footnotes

^1^ http://bio-bwa.sourceforge.net/bwa.shtml

^2^ http://www.ncbi.nlm.nih.gov/COG

^3^ https://www.genome.jp/kegg/

^4^ https://www.bic.ac.cn/imagegp/
